# Visfatin Is Regulated by Rosiglitazone in Type 2 Diabetes Mellitus and Influenced by NFκB and JNK in Human Abdominal Subcutaneous Adipocytes

**DOI:** 10.1371/journal.pone.0020287

**Published:** 2011-06-09

**Authors:** Kirsty C. McGee, Alison L. Harte, Nancy F. da Silva, Nasser Al-Daghri, Steven J. Creely, Christine M. Kusminski, Gyanendra Tripathi, Paul L. Levick, Manish Khanolkar, Marc Evans, Madhu V. Chittari, Vinod Patel, Sudhesh Kumar, Philip G. McTernan

**Affiliations:** 1 Unit for Diabetes & Metabolism, Clinical Sciences Research Institute, UHCW Trust, Walsgrave, Coventry, United Kingdom; 2 Biomarkers Research Program, Biochemistry Department, College of Science, King Saud University, Riyadh, Saudi Arabia; 3 Priory Hospital, BMI, Edgbaston, Birmingham, United Kingdom; 4 Heart Research Institute, Cardiff University, Wales, United Kingdom; 5 Diabetes Centre, George Eliot Hospital NHS Trust, Nuneaton, United Kingdom; Boston University, United States of America

## Abstract

Visfatin has been proposed as an insulin-mimicking adipocytokine, predominantly secreted from adipose tissue and correlated with obesity. However, recent studies suggest visfatin may act as a proinflammatory cytokine. Our studies sought to determine the significance of this adipocytokine and its potential role in the pathogenesis of T2DM. Firstly, we examined the effects of diabetic status on circulating visfatin levels, and several other adipocytokines, demonstrating that diabetic status increased visfatin*, TNF-α*** and IL-6*** compared with non-diabetic subjects (*p<0.05, **p<0.01, ***p<0.001, respectively). We then assessed the effects of an insulin sensitizer, rosiglitazone (RSG), in treatment naïve T2DM subjects, on circulating visfatin levels. Our findings showed that visfatin was reduced post-RSG treatment [vs. pre-treatment (*p<0.05)] accompanied by a reduction in HOMA-IR**, thus implicating a role for insulin in visfatin regulation. Further studies addressed the intracellular mechanisms by which visfatin may be regulated, and may exert pro-inflammatory effects, in human abdominal subcutaneous (Abd Sc) adipocytes. Following insulin (Ins) and RSG treatment, our *in vitro* findings highlighted that insulin (100 nM), alone, upregulated visfatin protein expression whereas, in combination with RSG (10 nM), it reduced visfatin*, IKKβ** and p-JNK1/2*. Furthermore, inhibition of JNK protein exacted a significant reduction in visfatin expression (**p<0.01), whilst NF-κB blockade increased visfatin (*p<0.05), thus identifying JNK as the more influential factor in visfatin regulation. Additional *in vitro* analysis on adipokines regulating visfatin showed that only Abd Sc adipocytes treated with recombinant human (rh)IL-6 increased visfatin protein (*p<0.05), whilst rh visfatin treatment, itself, had no influence on TNF-α, IL-6 or resistin secretion from Sc adipocytes. These data highlight visfatin's regulation by insulin and RSG, potentially acting through NF-κB and JNK mechanisms, with only rh IL-6 modestly affecting visfatin regulation. Taken together, these findings suggest that visfatin may represent a pro-inflammatory cytokine that is influenced by insulin/insulin sensitivity via the NF-κB and JNK pathways.

## Introduction

Following the recent isolation and characterization of visfatin, or pre-B-cell colony-enhancing factor (PBEF)/nicotinamide phosphoribosyltransferase (Nampt), as a novel adipocytokine, there has been a rapidly growing interest in this protein, its potential properties and subsequent role in the development of T2DM and obesity. Whilst the role of visfatin remains unclear, ablation of the gene proves fatal in visfatin knockout (KO) mice (−/−), during early embryogenesis [1]. These findings, in addition to the high level of evolutionary conservation of the visfatin gene [2], highlight its fundamental importance and emphasize how visfatin may play a key functional role in a variety of essential biological processes.

Previous studies have concentrated on the insulin mimetic properties of visfatin, due to the original work by Fukuhara and co-workers, with subsequent human studies noting increased circulating visfatin concentration in states of hyperglycemia and T2DM, which reduced with insulin treatment [3]–[6]. In contrast, several other studies debate the actual insulin-mimetic properties of visfatin, with such studies identifying a lack of association between visfatin and insulin resistance in humans [7]–[11], at either circulating or mRNA levels.

Following identification of the suggested insulin-mimetic properties of visfatin, studies have concurrently examined the role of insulin sensitizers, such as the thiazolidinediones (TZDs), on visfatin levels, which has resulted in the further generation of conflicting data. As such, treatment of non-diabetic (ND) subjects with rosiglitazone (RSG) has been shown to increase circulating visfatin levels [12], whilst, contrastingly, pioglitazone treatment has led to no apparent change in circulating levels in either T2DM or ND subjects [13], [14]. Assessment of visfatin mRNA levels in adipose tissue (AT), as a result of RSG treatment in the Otsuka Long Evans Tokushima Fatty (OLETF) rat (an animal model of T2DM with obesity), revealed increased mRNA expression levels in visceral fat depots [15], although decreased visfatin mRNA expression levels were reported in 3T3-L1 adipocytes [16]. In pioglitazone treated AT, visfatin mRNA expression in abdominal subcutaneous (Abd Sc) AT [16] or isolated adipocytes [17] remained unchanged. Furthermore, studies investigating the relationship between visfatin expression, adiposity and depot-specificity in human and rodent AT has also produced conflicting data [1], [7], [18]–[21].

The potential mechanisms involved in visfatin's activity in AT has remained largely under-studied beyond its ability to activate components of the insulin signaling pathway, such as insulin receptor substrates (IRS)-1/2 [1], [22], or PI3-kinase/Akt, by binding to the insulin receptor at a site distinct to insulin, itself [1]. Studies have begun to highlight visfatin's regulation of central transcription factors, such as nuclear factor (NF)-κB and activator protein (AP)-1 [23], [24]. This has addressed the potential for visfatin to elicit inflammatory responses [2], [24], [25], linked with elevated levels of pro-inflammatory factors, such as TNF-α and IL-6 [10], [25], [26], [27]. However, to date, the findings regarding visfatin's inflammatory role in the pathogenesis of T2DM, as well as the controlling mediators of visfatin regulation, remain unclear.

Therefore, the aims of this study were, firstly, to determine the systemic levels of visfatin in ND and T2DM subjects, as well as to establish the influence of RSG on circulating visfatin levels in newly diagnosed T2DM patients. Secondly, to further clarify whether an association exists between visfatin expression, increasing adiposity and depot-specificity in human AT (Abd Sc vs. Om AT), in addition to several other adipocytokines and factors implicated in the pathogenesis of the metabolic syndrome. Lastly, to investigate the potential inflammatory mechanisms via which visfatin may be regulated within the adipocyte, in addition to determining if visfatin regulates other pro-inflammatory adipocytokines, using *in vitro* analysis.

## Materials and Methods

### Ethics Statement

All studies were performed with the approval of the South Birmingham Ethics committee and Coventry and Warwickshire Ethics committee, with informed written consent obtained from all subjects prior to enrolment.

### Subjects

Fasting serum samples were collected from 34 ND and 30 T2DM subjects [ND BMI: 26.5(mean±SD) ±4.4 kg/m^2^; Age: 38.9±12.4 yrs; T2DM BMI: 37.2±9.0 kg/m^2^; Age: 51.3±12.6 yrs]. All T2DM patients had at least a 1 yr history of T2DM and remained on anti-diabetic therapy during sampling. For serum studies, anthropometric data were collected (Tables 1 & 2). Serum samples were assessed for glucose, insulin and cytokine levels, as detailed below. Fasting serum samples were also obtained from a cohort of newly diagnosed type 2 diabetic patients, who were treated for 10 weeks with RSG (4 mg twice daily), [BMI: 35.5±6.0 kg/m^2^; Age: 55.4±11.1 yrs, n = 12]. In addition, matched Abd Sc and Om whole AT samples were obtained from a human ND population (n = 47, age: 43.8±8.4 yrs [mean±SD]; BMI: 29.2±9.6 kg/m^2^) undergoing elective surgery, for the purpose of matched pair protein analysis. These subjects had no medical conditions (i.e. hypertension, CVD, thyroid disorders, renal disorders, diabetes or chronic pain conditions). None of the subjects were on endocrine therapy (i.e. hormonal replacement therapy (HRT), tamoxifen, steroids, thyroxine) anti-inflammatory therapy (aspirin, cyclooxygenase-2 inhibitors), statins, thiazolidinediones (TZDs) or antihypertensive therapy.

**Table 1:** Clinical and Biochemical Characteristics of T2DM Subjects Compared with Control Subjects.

	Control (n = 34)	T2DM (n = 30)	*P* value
**Age, yrs**	38.9±12.4	51.3±12.6	0.00021
**BMI, kg/m^2^**	26.5±4.4	37.2±9.0	<0.0001
**Sex (M∶F)**	12∶22	24∶6	NA
**Visfatin, log ng/mL**	11.39 (9.64, 18.97 )	23.01 (11.77, 28.28)	0.008
**Insulin, (median IU/mL (min, max)**	11.4 (2.8, 18.1)	11.5 (3.5, 25.7)	N/S
**Glucose mmol/L**	5.47±0.77	8.36±2.66	0.0009
**HOMA-IR**	2.91±1.53	4.16±1.68	0.043
**TNF-α, pg/mL**	11.11±3.28	16.9±16.5	0.00038
**IL-6, pg/mL**	4.61±2.32	7.03±2.42	0.00014
**sCD14, million IU/mL**	1.45±0.40	2.8±1.01	<0.0001
**Leptin, log pg/mL**	15.68 (5.89, 33.79)	22.79 (12.18, 45.72)	N/S

Data are presented as mean (±SD), unless otherwise stated. Non-parametric data have been log transformed in which case they are presented as mean (interquartile range).

**Table 2:** Clinical and Biochemical Characteristics of T2DM Subjects Pre and Post 10 week Rosiglitazone Treatment.

	Pre RSG (n = 12)	Post RSG (n = 12)	*P* value
**Age, yrs**	55.4±11.1	N/A	N/A
**BMI, kg/m^2^**	35.5±6.0	37.6±6.7	N/S
**Sex (M∶F)**	1∶1	N/A	NA
**Visfatin, ng/mL**	10.21±4.23	7.45±4.53	0.04
**Insulin, IU/mL**	15.79±7.58	8.37±4.02	0.017
**Glucose, log mmol/L**	7.97 (7.49, 12.45)	6.58 (5.62, 8.43)	0.018
**HOMA-IR**	7.87±3.96	3.05±2.03	0.009
**IL-6, log pg/mL**	3.43 (2.25, 7.09)	4.10 (2.80, 5.11)	N/S
**Adiponectin, log mg/mL**	9215.15 (6460.90, 11771.75)	12083.75 (6478.65, 26585.25)	0.04
**Leptin, log pg/mL**	23.43 (13.69, 60.70)	26.37 (13.76, 49.81)	N/S
**sCD14, log million IU/mL**	2.10 (1.59, 3.21)	2.35 (1.86, 3.15)	N/S

Data are presented as mean (±SD). Non-parametric data have been log transformed in which case they are presented as mean (interquartile range).

### Isolation of Mature Adipocytes

Abd Sc AT was digested in collagenase (2 mg/mL; Worthington Biochemical, USA) in order to isolate the mature adipocytes, as previously described [28]. Following isolation, adipocytes were maintained in phenol red-free DMEM:Ham's F-12 medium containing 15 mmol/L glucose, penicillin (100 U/mL) and streptomycin (100 µg/mL) with the various treatment regimens, described below.

### Treatment of Abd Sc Adipocytes with Visfatin, TNF-α, IL-6, JNK/NF-κB Inhibitors, Insulin and/or RSG

Mature adipocytes in 1 mL aliquots (containing approximately 500,000 adipocytes) were maintained in 25 cm^2^ flasks containing 5 mL phenol red-free DMEM:Ham's F-12 medium for 48 hours (hrs), then treated with recombinant human (rh) insulin alone (100 nM; Sigma UK) with optimal concentrations determined in previous studies [29]–[32], or in combination with RSG, (10 nM; GlaxoSmithKline, UK), rh visfatin alone (1, 100 nM; Phoenix Pharmaceuticals, USA), or combined with 10 nM RSG, rh TNF-α (1, 5, 10 ng/mL; Biosource International Inc, Belgium) and rh IL-6 (1, 5, 10 ng/mL; Sigma-Aldrich, UK) for 48 hrs. For inhibition studies, adipocytes were incubated for 24 hrs with NF-κB inhibitor (NF-κB: SN50, 50 µg/mL; Calbiochem, UK) and/or c-Jun N-terminal Kinase (JNK) inhibitor (SP600125, 10 µM; A.G. Scientific, Inc., San Diego, CA). For all treatments, untreated adipocytes maintained in medium for 48 hrs were used as controls, unless otherwise stated. Viability of adipocytes was assessed using trypan blue (Sigma) as previously described [28]. Furthermore, adipocytes remained insulin sensitive, as demonstrated by previous analysis of multiple adipocytokines, including TNF-α, which displayed changes in levels with increasing insulin concentration that otherwise would have remained unchanged within insulin resistant cells [29]–[32]. Following incubation (37°C/5%CO_2_), adipocytes and their conditioned media were separated by centrifugation (360 *g* for 2 minutes). Conditioned media were removed, aliquoted and stored at −80°C for future use. Adipocyte protein was extracted using a radio-immunoprecipitation (RIPA) buffer [29], then stored at −80°C, until required for analysis.

### Analysis of Serum Adipokine and Hormone Levels

Serum visfatin levels were assayed using a commercially available Visfatin C-terminal human EIA Kit (Phoenix Pharmaceuticals, Inc. USA; Intra-assay: <5%; Inter-assay: <14%). In addition, serum levels were also assessed to detect levels of TNF-α, IL-6 (BenderMedSystems, Vienna, Austria), sCD14 (R&D Systems, Abingdon, UK), leptin, adiponectin and insulin (Linco Research, St. Charles, MO). Serum levels were analyzed using a solid phase enzyme-linked immunosorbant assay (TNF-α CV intra-assay 6·0%, inter-assay 9·3%; IL-6 CV intra-assay 6·2%, inter-assay 7·0%; sCD14 CV intra-assay 5.4%, inter-assay 6.3%; Leptin CV intra-assay 2.6%, CV inter-assay 3.8±3.4%; insulin CV intra-assay 5·96%, inter-assay 10·3±0·9%. Fasting glucose levels were analyzed using a glucose oxidase method (YSL 200 STAT plus). Insulin resistance (HOMA-IR) was derived using a HOMA equation [33]. Analyses were performed on log transformed data in cases where serum levels were not normally distributed.

### Assessment of Adipocytokine Secretion from Visfatin Treated Abd Sc Adipocytes

Conditioned media from cultured control (untreated) Abd Sc adipocytes and visfatin treated adipocytes were assayed using commercially available human ELISAs for resistin (Phoenix Europe GmbH, Germany) (Intra-assay CV, <3%; Inter-assay CV, <10%; Minimum Detectable Concentration (sensitivity of assay: 0.016 ng/mL)), TNF-α (0.1 ng/mL) and IL-6 (16 pg/mL). In addition to this, conditioned media from cultured control untreated Abd Sc adipocytes and insulin treated adipocytes, were assayed for IL-6.

### Protein Determination & Western Blot Analysis

Protein was extracted from isolated adipocytes using a RIPA buffer (150 mmol/L NaCl, 1.0% IGEPAL® CA-630, 0.5% sodium deoxycholate, 0.1% SDS, and 50 mmol/L Tris), as previously documented [29]. Protein concentrations were quantified using the Bio-Rad DC (Detergent Compatible) protein assay kit [34]. Western blot analysis was performed using a standard method previously described [32]. In brief, 25–30 µg of protein was loaded onto a 10% polyacrylamide gel (Geneflow Ltd., Fradley, UK), under reducing conditions, then a human visfatin polyclonal antibody (1∶1000; Biogenesis Ltd., Poole, UK), was utilized. Polyclonal phosphospecific JNK1 and 2 (p-JNK1/2), NF-κB and IKKβ antibodies (1∶1750, Biosource International; 1∶250 & 1∶250, Calbiochem, UK, respectively) were also utilized. Blots were developed using an anti-rabbit (Visfatin, 1∶10,000; p-JNK1/2, 1∶60,000), or anti-mouse (NF-κB, 1∶6000; IKKβ, 1∶10,000) horseradish-peroxidase secondary antibody (The Binding Site, UK). Equal protein loading was confirmed using β-actin (Cell Signaling, UK) expression (Primary antibody, 1∶1000; Secondary antibody, 1∶10,000). A chemiluminescent detection system ECL/ECL^+^ (GE Healthcare, Amersham Biosciences, Little Chalfont, UK) allowed visualization following exposure to X-ray film. Intensity was then determined using densitometry (GeneTool software, Syngene, UK).

### Immunohistochemical Analysis

Immunohistochemical analysis of normal kidney and AT was carried out according to previously described methods [28]. Human Abd Sc and Om AT samples were incubated with a polyclonal antibody to human visfatin (Phoenix pharmaceuticals, California USA) at a concentration of 1∶250. Human kidney tissue has been previously reported to express visfatin [35], [36] and was therefore utilized as a positive control for visfatin expression. All tissue slides were developed using a peroxidase substrate kit and counterstained with Mayer's hematoxylin.

### Extraction of AT RNA and Quantitative Real-Time Polymerase Chain Reaction

RNA was extracted from whole Abd Sc and Om AT, as well as differentiated pre-adipocytes, mature isolated Om and Abd Sc adipocytes (RNeasy Lipid Tissue Mini Kit, Qiagen, UK). RNA extraction was followed by a DNase digestion step to remove any contaminating genomic DNA. RNA (200 ng) from each sample, alongside human macrophage RNA (Yorkshire Biosciences, UK), was reverse transcribed using a reverse transcriptase (SuperScript III, Invitrogen, UK) and random hexamers in 20 µL reaction volumes, according to the manufacturer's instructions. Messenger RNA levels were determined using an ABI 7500 real time PCR Sequence Detection system, as previously outlined [37].

Visfatin gene expression was quantified using a commercially available TaqMan™ Gene Expression Assay (Accession number: NM_005746.2, Applera, Cheshire, UK) and reactions were carried out in 25 µL volumes on 96 well plates, in a reaction buffer containing TaqMan Universal PCR Master Mix and 1 µL cDNA. All reactions were multiplexed with the housekeeping gene 18S, provided as a pre-optimised control probe (Applera, Cheshire, UK) allowing data to be expressed as delta cycle threshold (ΔCt) values (ΔCt = Ct of 18S gene subtracted from Ct of gene of interest) in order to correct for differences in the efficiency of reverse transcription. Measurements were carried out in triplicate. All statistics were performed at the ΔCt stage in order to exclude potential bias due to averaging of data transformed through the equation ^2−ΔΔ^Ct. Statistical analysis was performed using a paired t-test (SPSS Inc. 15.0, Woking, UK), unless otherwise stated.

### Analysis of Circulating Visfatin Levels

Serum visfatin levels were assayed using a Visfatin C-terminal human EIA Kit (Phoenix Pharmaceuticals, Inc. USA). Intra-assay: <5%; Inter-assay: <14%. TNF-α, IL-6 and insulin levels were analyzed using a solid phase enzyme-linked immunosorbant assay. TNF-α CV intra-assay 6·0%, inter-assay 9·3%; IL-6 CV intra-assay 6·2%, inter-assay 7·0%; insulin CV intra-assay 5·96%, inter-assay 10·3±0·9%. Fasting glucose levels were analyzed using a glucose oxidase method (YSL 200 STAT plus). Insulin resistance (HOMA-IR) was derived using a HOMA equation [33].

### Statistical Analysis

Protein expression data were compared using a student's t-test, unless otherwise stated, and are presented as mean relative fold difference ± SEM, unless otherwise stated. Data from mRNA studies underwent analysis, as detailed in the quantitative RT-PCR section. Correlations were calculated using a Pearsons' Correlation Coefficient test. The threshold for significance was *p*<0.05. All analysis was carried out using the SPSS version 15 (SPSS Inc. Chicago) software package.

## Results

### Immunohistological Staining of Visfatin in AT

**Figure 1:**
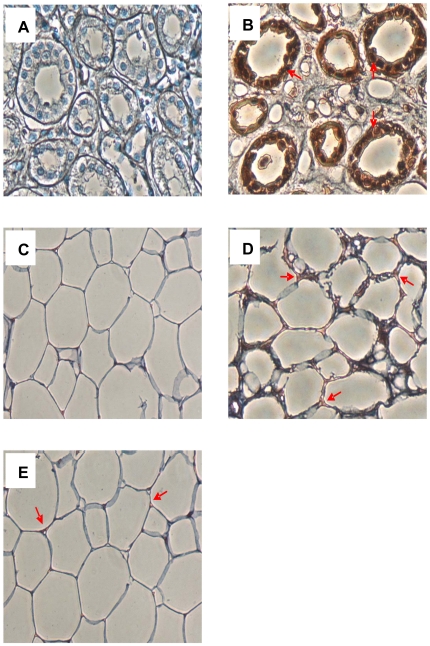
A–E. Photographs of Immunological Staining of Visfatin Protein. This figure shows the immunological staining of visfatin in (A) human kidney negative (primary antibody omitted); (B) human kidney positive control; (C) human AT PBEF negative (primary antibody omitted); (D) human Om AT; (E) human Abd Sc AT. Red arrows highlight areas of red staining to show the presence of the visfatin protein.

Positive immuno-staining of visfatin (Figure 1) in the cytoplasm and cell membrane was confirmed in both Om (D) and Abd Sc (E) AT. Omission of the primary visfatin antibody (C) did not reveal any positive staining. Human kidney tissue was used as a control, also undergoing primary antibody omission (A) and positive immunohistological staining for visfatin (B).

### Circulating Visfatin Levels in Non-Diabetic and T2DM Subjects

Serum visfatin levels were significantly higher in T2DM subjects in comparison with ND subjects (**p<0.01, Table 1). Pearson coefficient correlations were carried out to assess the relationship between circulating visfatin levels and increasing age or BMI, and a moderate, positive correlation was observed between visfatin and BMI in ND control subjects (*p<0.05). A positive correlation was also observed between visfatin and HOMA-IR (*p<0.05). Serum visfatin levels did not correlate with any other parameters. Serum analysis revealed a significant elevation in TNF-α (***p<0.001), IL-6 (***p<0.001), glucose (***p<0.001), HOMA-IR (*p<0.05) and sCD14 (***p<0.001) levels in T2DM subjects when compared against ND subjects (Table 1). No significant differences in insulin concentration were observed between T2DM and ND controls (Table 1).

### Effect of the Insulin Sensitizer, RSG, on Circulating Visfatin Levels in Newly Diagnosed T2DM Patients

Visfatin levels were measured in newly diagnosed, treatment naïve, T2DM patients pre- and post- 10 week treatment with 10 nM RSG. A significant reduction in serum visfatin was observed in post-RSG treated T2DM subjects when compared with pre-RSG treatment (*p<0.05, n = 12, Table 2). Serum visfatin concentration did not correlate with any clinical or biochemical parameters in this cohort (Table 2). Serum analysis revealed a significant decrease in the circulating levels of insulin (*p<0.05), glucose (*p<0.05) and HOMA-IR (**p<0.01), following RSG treatment, whilst an increase was observed with adiponectin (*p<0.05). No correlations were observed between any of the other clinical or biochemical parameters (Table 2).

### Visfatin Protein and Gene Expression in Relation to Adiposity and Depot Specificity

**Figure 2:**
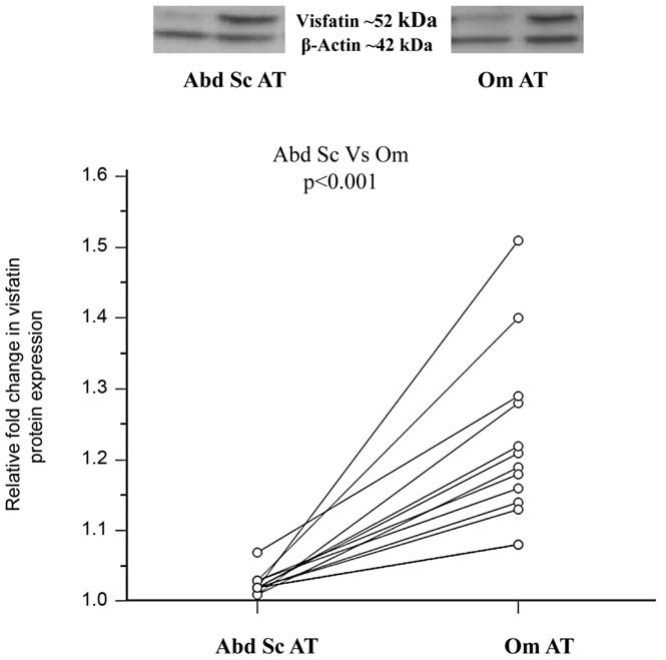
Visfatin Protein Expression in Relation to Adiposity and Depot Specificity. This figure shows the mean relative fold protein expression of visfatin in omental (Om) versus abdominal (Abd) adipose tissue depots in matched pairs, from a cohort of lean and obese subjects, where the Abd Sc depot has been standardized. The representative Western blots are shown above. Lean samples: n = 7 & Obese samples: n = 6; p-values: p<0.001.

The investigation of visfatin protein expression in relation to depot specificity in human AT identified that visfatin protein was significantly higher in Om AT, when compared with paired Abd Sc AT, independent of BMI (Lean: n = 7; Obese: n = 6, total: n = 13, ***p<0.001, Figure 2). Furthermore, an association between increasing adiposity and visfatin expression in human Abd Sc adipocytes and AT was not observed at either protein or mRNA level (data not shown). Lastly, differentiated pre-adipocytes and mature isolated adipocytes (Om and Abd Sc) demonstrated the presence of visfatin mRNA in isolated adipocytes: differentiated adipocytes ΔCt = 5.7±1.35(SEM), Om adipocytes ΔCt = 11.02±0.125, Abd Sc adipocytes ΔCt = 11.16±0.077. The mRNA visfatin level in human macrophages was comparable to that of the mature Om and Abd Sc adipocytes (ΔCt = 10.93±0.074), whilst visfatin expression was significantly lower in Om and Sc stromal cells, respectively, ΔCt = 12.75±0.175, Sc stromal ΔCt = 16.1±0.08 (p<0.05).

### Effect of Recombinant Human (rh) Insulin on Visfatin Protein Expression

**Figure 3:**
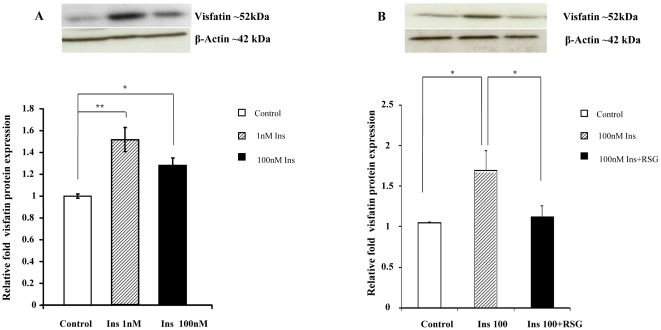
A&B. The Effect of Insulin Alone and in Combination with RSG on Visfatin Protein Expression in Isolated Adipocytes. These figures show the mean relative protein expression (±SEM) of visfatin in isolated human adipocytes treated with insulin (1 nM and 100 nM, respectively, Figure 3A) and insulin in combination with RSG (Figure 3B), compared with control (untreated adipocytes) as an arbitrary value of 1. The representative Western blots are shown above (n = 4, p-values: p<0.05*, p<0.01**).

The influence of increasing rh insulin concentration (Ins 1 nM, 100 nM) on visfatin in isolated human Abd Sc adipocytes was investigated. From this, it was observed that visfatin protein expression significantly increased with both concentrations of insulin, as compared with untreated controls (Control vs. Ins 1 nM, **p<0.01; Control vs. Ins 100 nM, *p<0.05, Figure 3A). The effect of insulin alone and in combination with RSG on visfatin in isolated Abd Sc adipocytes was further examined and determined that RSG 10 nM/Ins 100 nM significantly reduced visfatin protein expression compared with insulin alone (100 nM) 48 hrs post treatment (*p<0.05, Figure 3B).

### Effect of rh Insulin and RSG on JNK1/2 and IKKβ Protein Expression

**Figure 4:**
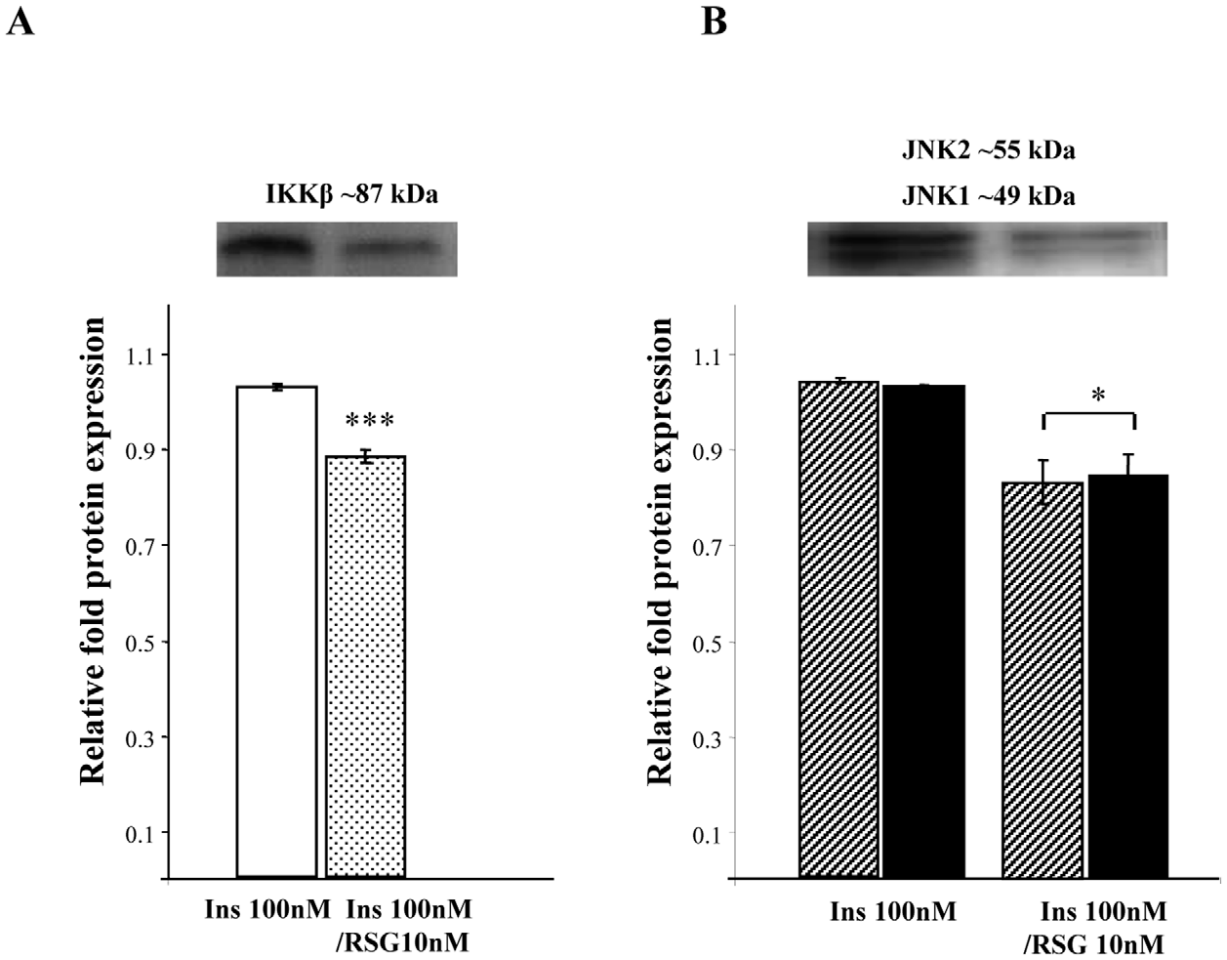
A&B. The Effects of Insulin and RSG on IKKβ and JNK1/2 Protein Expression. These figures shows the effect of insulin, alone (100 nM), and insulin in combination with RSG (Ins 100 nM/RSG 10 nM) on the mean relative fold protein expression (± SEM) of IKKβ (Figure 4A) and JNK 1/2 (Figure 4B). The representative Western blots are shown above (n = 4, p-values: p<0.05*, p<0.001***).

In mature Abd Sc adipocytes, treatment with Ins 100 nM/RSG 10 nM was shown to significantly reduce IKKβ and JNK 1/2 protein expression (*p<0.01 and *p<0.05, respectively) compared with adipocytes treated with insulin alone (100 nM) (Figures 4A and 4B).

### Effect of JNK and NF-κB Inhibition on Visfatin Protein Expression in Human Isolated Abd Sc Adipocytes

**Figure 5:**
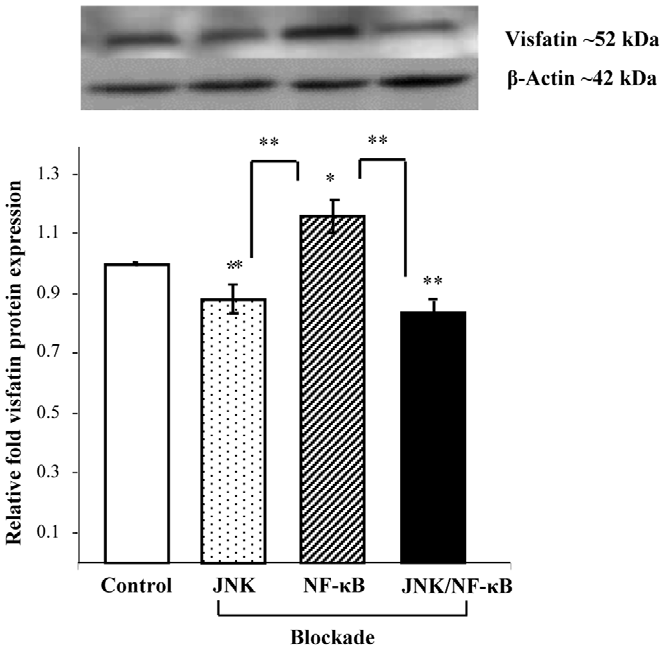
The Effects of JNK and NF-κB Inhibition on Visfatin Protein Expression in Human Adipocytes. This figure shows the effect of JNK and NF-κB blockade, alone and in combination, on the mean relative fold protein expression (±SEM) of visfatin in isolated Abd Sc adipocytes compared with control (untreated adipocytes), given an arbitrary value of one. The representative Western blots are shown above (n = 4, p-values: p<0.05*, p<0.01**).

The effects of NF-κB and JNK inhibition on visfatin protein expression were investigated in mature isolated Abd Sc adipocytes. These studies identified that visfatin expression was significantly reduced with JNK inhibition alone (*p<0.05, Figure 5). Similarly, when NF-κB was inhibited, visfatin expression was markedly increased in comparison with control (*p<0.05, Figure 5). Interestingly, when both JNK and NF-κB were inhibited, visfatin expression was significantly reduced when compared with control Abd Sc adipocytes (**p<0.01, Figure 5).

### Effect of rh TNF-α and IL-6 on Visfatin Protein Expression in Human Isolated Abd Sc Adipocytes

In human adipocytes, rh IL-6 was observed to significantly upregulate visfatin protein expression (rh IL-6: Control: 1.0±0.01 (relative fold expression, 1 nM: 1.15±0.05, *p<0.05). However, no significant difference was observed with rh TNF-α treatment (data not shown).

### Effect of rh Visfatin and RSG on NF-κB and pJNK1/2 Protein Expression

In Abd Sc adipocytes treated with rh visfatin (100 nM) alone, or in combination with RSG (10 nM), no significant effect on protein expression of either NF-κB or phosphospecific JNK1/2 was observed (data not shown).

### Effect of Visfatin and Insulin on Adipocytokine Secretion from Mature Isolated Adipocytes into Conditioned Media

Incubation of mature isolated adipocytes with rh visfatin, and subsequent analysis revealed visfatin to have no influence on adipocyte secretion of TNF-α, IL-6 or resistin. Incubation of mature isolated adipocytes with rh insulin identified increasing insulin concentration to upregulate IL-6 secretion (Control: 2.5±0.3 pg/mL versus (vs.) Ins 1 nM: 12.0±1.1 pg/mL, **p<0.01; control vs. Ins 10 nM: 15.0±4.2 pg/mL, ***p<0.001; control vs. Ins 100 nM: 30±3.6 pg/mL ***p<0.001) (data not shown).

## Discussion

This present study contributes to our understanding of the dual nature of visfatin through insulin and pro-inflammatory mechanisms. Our current *in vivo* findings identified that T2DM patients exhibited significantly higher circulating visfatin levels in comparison with ND subjects, in accordance with previous findings [3], [4], but levels decreased upon treatment with RSG. Furthermore, our *in vitro* studies demonstrated that insulin upregulated visfatin protein expression, which was subsequently attenuated by the insulin sensitizer RSG. The action of insulin on visfatin expression may occur indirectly, in part, through the up-regulation of IL-6 in Abd Sc AT as noted by our studies. These studies also established that visfatin appears to be regulated via both NF-κB and c-Jun Kinase. Collectively, both the *in vivo* and *in vitro* findings suggest a role for NF-κB and c-Jun Kinase in the stimulation of pro-inflammatory cytokines including visfatin, IL-6 and TNF-α from human Abd Sc adipocytes; with IL-6 acting as a partial positive mediator for visfatin expression. This further implicates human AT as an important site for the progression of low-grade inflammation in T2DM.

The role of visfatin in relation to increasing adiposity has been the subject of much debate over recent years [1], [7], [9], [19], [38]. Our present *in vivo* studies examined this relationship and found a significant, positive correlation between increasing BMI and circulating visfatin levels in the ND subjects, albeit a modest one. This limited effect may have been a result of the narrow range of BMI within the control cohort. Furthermore, the T2DM subjects exhibited significantly higher serum levels of visfatin than their control counterparts. As such, our data suggest that visfatin levels are influenced by BMI but that in the diabetic state, BMI ceases to prevail as the major influential factor. Further analysis, examining visfatin expression from *ex vivo* human AT, established no change in visfatin with adiposity, at either the gene or protein level in Abd Sc and Om tissue - a finding supported by a recent study carried out in rodents [18]. However, depot specific analysis of visfatin showed significantly higher protein levels in Om AT when compared with Abd Sc, confirming the original findings by Fukuhara *et al* [7]. Whilst the *ex vivo* visfatin tissue findings appear in conflict with the *in vivo* findings, this may be further complicated as systemically the production of visfatin may be derived from other sources, such as the liver, which may affect the specific influence of AT [39], [40]; highlighting the complexity of the interplay between AT and other tissues, *in vivo*.

Our findings also showed RSG to down-regulate circulating visfatin concentrations, paralleled with a significant reduction in insulin levels, *in vivo*. This was in contrast to findings with pioglitazone where visfatin levels remained unaffected. Such a difference may have occurred due to several factors including the duration of treatment, the characteristics of the diabetic cohort and the different properties of the various TZDs [17]. However, based on our current data, further *in vitro* analysis evaluated the influence of insulin and RSG on visfatin in cultured Abd Sc AT. Our previous *in vitro* studies on isolated human adipocytes observed that insulin stimulated the release of various pathogenic adipocytokines, whilst RSG inhibited their release [29], [31], [32]. Our current studies demonstrated that insulin significantly increased visfatin protein expression, with lower levels producing a greater response, whilst RSG, in combination with insulin, significantly lowered visfatin protein expression compared with cells treated with insulin alone. Therefore, both the *in vivo* and *in vitro* data suggest visfatin is regulated by insulin. The observation that higher insulin levels lead to lower visfatin expression indicates the potential for a negative feedback loop. We may, therefore, speculate that the presence of high insulin levels in the insulin sensitive cell negates the need for high levels of visfatin and therefore suppresses its production. In this instance, visfatin may, potentially, be a marker of insulin resistance, where the cell perceives lower levels of insulin and greater visfatin production arises, as a result. Hence RSG may be acting directly on visfatin, exerting some of its anti-diabetic activities via the reduction of visfatin levels, in addition to other adipocytokines, or indirectly due to its insulin sensitizing effects on the cell.

At present, there are limited data regarding visfatin regulation and the mechanisms through which it may act in human AT. Previous studies have observed treatment of 3T3-L1 adipocytes with either rh TNF-α or rh IL-6 to decrease visfatin mRNA expression levels [26], [27]. Therefore, our studies addressed whether rh TNF-α and/or rh IL-6 could alter visfatin protein expression levels in cultured human Abd Sc adipocytes. The results of our *in vitro* experiments suggest a potential mechanism through which visfatin may be regulated and indicate a possible cause for elevated visfatin levels in T2DM subjects. The findings showed that increasing rh IL-6 concentration modestly upregulated visfatin protein expression in Abd Sc adipocytes, comparable with other studies [8], [10], [25]. In addition to this, rising insulin concentrations increased IL-6 secretion using the same *in vitro* system. Therefore, taken together, the findings suggest that, in the diabetic state, high circulating insulin levels may indirectly contribute to elevated visfatin levels via upregulation of IL-6. Thus, whilst previous studies have identified that visfatin activates insulin's intracellular signalling cascade [1], [19], visfatin may be upregulated in the event of insulin signalling impairment - an impairment that may result, partly, from the actions of IL-6 altering its functionality, as well as through the effect of hyperinsulinaemic conditions on the tissue.

In addition to this, our current *in vitro* studies have identified a potential pathway through which visfatin may be regulated in the adipocyte, as noted through the attenuating effects of RSG on p-JNK 1/2 and NF-κB protein expression. To delineate these, further studies examined the influence of these intracellular signalling molecules through the use of JNK and NF-κB inhibitors in isolated human Abd Sc adipocytes. These *in vitro* studies indicated that the use of a JNK inhibitor reduced visfatin protein expression compared with untreated controls, whilst NF-κB inhibition led to increased visfatin protein expression. Furthermore, a combination of both inhibitors resulted in the most significant reduction in visfatin protein expression, compared with untreated controls, in a similar fashion to the effects noted by JNK inhibition, alone, suggesting that JNK has an important regulatory influence on NF-κB in the mediation of visfatin expression. To date, few studies have investigated the potential relationship between JNK/NF-κB and visfatin. However, genomic analysis of the visfatin gene has identified regulatory elements of NF-κB and AP-1 [41], with another study, in human leukocytes, noting that visfatin is upregulated by NF-κB p65 binding activity [25]. Furthermore, both NF-κB and AP-1 have been shown to upregulate visfatin in human amniotic epithelial cells [23]. These findings, therefore, suggest that JNK and NF-κB pathways are involved in visfatin regulation in the human adipocyte, with JNK exerting a more influential role than that of the NF-κB pathway. These studies also demonstrate that the JNK and NF-κB pathways are not the sole mechanisms governing visfatin regulation and, as with many other adipocytokines, a delicate interplay exists between various intracellular signalling mechanisms. Hence, further studies are required to delineate, in full, the intracellular pathways that govern visfatin.

Although visfatin is presently the focus of intense study, to date, many of the current findings show inconsistencies with regard to visfatins role in the pathogenesis of obesity-mediated T2DM and the metabolic syndrome. However, it is evident that significant associations between visfatin and inflammatory disease states do exist [42]–[44]. This, perhaps, is not surprising considering AT is comprised of a variety of cells other than adipocytes, including macrophages and stromal cells, which are also known to express visfatin. However, this study has highlighted the role of the adipocyte within AT in relation to visfatin, whilst other studies have focused on macrophage function in this capacity [45], [46]. The dichotomous nature of the adipocyte, regarding metabolism and immunity, combined with it being the major constituent of AT, makes determining the significance of visfatin in adipocyte biology of fundamental importance in elucidating its function.

Our present findings demonstrate that rh visfatin does not influence inflammatory adipocytokines, such as IL-6, TNF-α and resistin, implicating other factors in visfatin mediated activity. Our studies do appear to cast light on the mechanisms governing visfatin's expression in human adipocytes, with these findings suggesting visfatin regulation by Ins/RSG and IL-6 is mediated, in part, through JNK and NF-κB activation. This interaction represents a significant insight into visfatin's metabolic regulation in human AT and further reflects how visfatin represents a relevant adipocytokine in metabolic disease. Furthermore, our findings highlight the reduction of visfatin, in combination with several other cytokines, during RSG treatment of T2DM patients. This, in conjunction with our previous data, also showing RSG to lower other pathogenic factors [29], [31], [32], represents a novel finding that may underlie the clinical, metabolic and vascular effects of RSG. Finally, although visfatin's role in the pathogenesis of obesity and T2DM, as yet, remains somewhat controversial, it seems that visfatin does not appear to be a mere bystander and its pathological, biological and functional state and influence, may depend on the metabolic state in which it is found.
